# Ductular Reaction and Liver Regeneration: Fulfilling the Prophecy of Prometheus!

**DOI:** 10.1016/j.jcmgh.2022.11.007

**Published:** 2022-11-24

**Authors:** Satdarshan P. Monga, Kari Nejak-Bowen

**Affiliations:** 1 Division of Experimental Pathology, Department of Pathology, University of Pittsburgh Medical Center and University of Pittsburgh School of Medicine, Pittsburgh, Pennsylvania; 2 Division of Gastroenterology, Hepatology and Nutrition, Department of Medicine, University of Pittsburgh Medical Center and University of Pittsburgh School of Medicine, Pittsburgh, Pennsylvania; 3 Pittsburgh Liver Research Center, University of Pittsburgh Medical Center and University of Pittsburgh School of Medicine, Pittsburgh, Pennsylvania

**See counterpoint on** **page 803** **.**

Many acute and chronic liver injuries exhibit histologically as proliferating cholangiocytes, commonly referred to as ductular reaction. The origin of the cells comprising the ductular reaction is dependent on the injury (hepatocyte vs cholangiocyte) and capability of these 2-liver epithelial or “hepithelial” cells to divide and replace injured cells. The role of ductular reaction in hepatobiliary injury versus repair remains debated. Although ductular reaction has been shown to be a source of proinflammatory and profibrogenic factors, it has also been shown to contribute toward maintaining hepatobiliary function during injury. In fact, reducing ductular reaction has been shown to exacerbate, whereas promoting it could alleviate liver injury. We discuss some of the studies that highlight various mechanisms through which ductular reaction plays a beneficial role in modulating hepatobiliary health during injury and eventually discuss how regulating such process may have a therapeutic benefit.

Ductular reaction is a common hallmark of many liver pathologies including cholangiopathies, viral hepatitis, and alcoholic and nonalcoholic fatty liver diseases.^1^^,^^2^ Although variable in its histologic appearance, ductular reaction is caused usually by proliferation of cholangiocytes or because of transdifferentiation of hepatocytes into cholangiocytes. The ductular reaction can manifest as appearance of multiple small ducts with well- to ill-defined lumen, appearance of multilayered ductular structures, or as isolated small cells with biliary markers interspersed in the hepatic parenchyma beyond the portal mesenchyme, referred to as invasive ductular reaction.^3^ Preclinically, such variability in ductular _reaction can be seen in response to bile duct ligation, diets that are choline-deficient ethionine-supplemented (CDE), or containing 3,5-diethoxycarbonly-1, 4-dihydrocollidine, among other protocols. Such variable ductular reaction can also be seen in patients with alcoholic hepatitis, primary sclerosing cholangitis, primary biliary cholangitis, viral hepatitis, and even nonalcoholic steatohepatitis cases, and the overall significance remains debated and not completely clear.^1^^,^^2^

Why would ductular reaction occur in such variable hepatic pathologies in the first place? Liver is unique in its ability to regenerate and intriguingly, all constituent cells of the liver participate in the process.^4^ Hence a simple explanation for the ductular reaction in many preclinical models and in clinical scenarios is that ductular reaction is a reparative response to cholangiocyte injury. When cholangiocytes are the target of an insult that may be genetic, immunologic, iatrogenic, infectious, obstructive, or idiopathic, some cholangiocytes die, whereas the others proliferate and expand to repair the injured bile ducts. Thus, in a subset of pathologies, ductular reaction may be a valuable component of the reparative response to replace dying cholangiocytes. It is conceivable that such repair may be transient and nonperpetuating, especially if an injury is self-limited or is treated. However, if injury to the cholangiocytes persists, ductular reaction lingers to continually replace dying cholangiocytes and may manifest as persistent, variable, and atypical ductular reaction. Such forms of ductular reaction may be an attempt to generate de novo collaterals to drain bile, or merely generate “ectopic” cholangiocytes, to compensate for lost cholangiocyte mass and perform some functions as discussed next.

Cholangiocytes line the intrahepatic bile ducts, which serve as a conduit to transport bile from the biliary canaliculi to the extrahepatic biliary tree and small intestines. In this manner, bile, which contains relatively toxic bile acids among other components, stays sequestered from other cells within the hepatic parenchyma. Cholangiocytes also regulate the composition and flow of bile through absorption and secretion of ions, especially HCO_3_-, water, glucose, and others, into the bile.^5^ Unconjugated bile acids can passively diffuse into cholangiocytes and to the hepatocytes via cholehepatic shunting, whereas conjugated bile acids can be absorbed by apical Na^+^-dependent bile acid transporter. Cholangiocytes express several xenobiotic metabolism enzymes, such as CYP2E1 and CYP1A2, and also participate in the cholesterol metabolism among many other functions.^6^^,^^7^

Other than providing cells to replace dead cholangiocytes and “patch” leaking bile ducts, the ductular reaction could promote repair in an injury setting in 3 different ways. First, in the setting of intrahepatic cholestasis, a ductular reaction that is observed as number of small duct-like structures that are mostly lined by a single layer of cholangiocytes and contain lumen, may really serve as collaterals to rid the liver of bile. It is likely that these de novo mini ductular structures serve as channels to drain bile into existing intact remnant intrahepatic bile ducts that may be relatively spared by an insult. Indeed, Kok et al^8^ have shown that when animals were fed such diets as CDE, the depth and complexity of the intrahepatic tree increases drastically, with terminal ducts now almost encroaching the hepatic parenchyma in the pericentral zone. As hepatocytes succumb to such diets as CDE and there is an ensuing collapse of hepatocyte-lined biliary canaliculi, such ductular reaction may be the only way to collect bile produced by remnant hepatocytes so that it can be eventually transported into the intestines.

Second, the increase in cholangiocyte mass irrespective of the presence of a functional lumen may contribute to some of the static functions of cholangiocytes, such as in xenobiotic or drug metabolism and cholesterol metabolism. Thus, as bile ducts succumb to pathologic insults, the ductular reaction, even without a patent lumen, may assist in maintaining homeostasis by helping regulate the content and toxicity of bile acids produced in hepatocytes and performing limited but important metabolic functions.^2^^,^^6^

**Figure 1:**
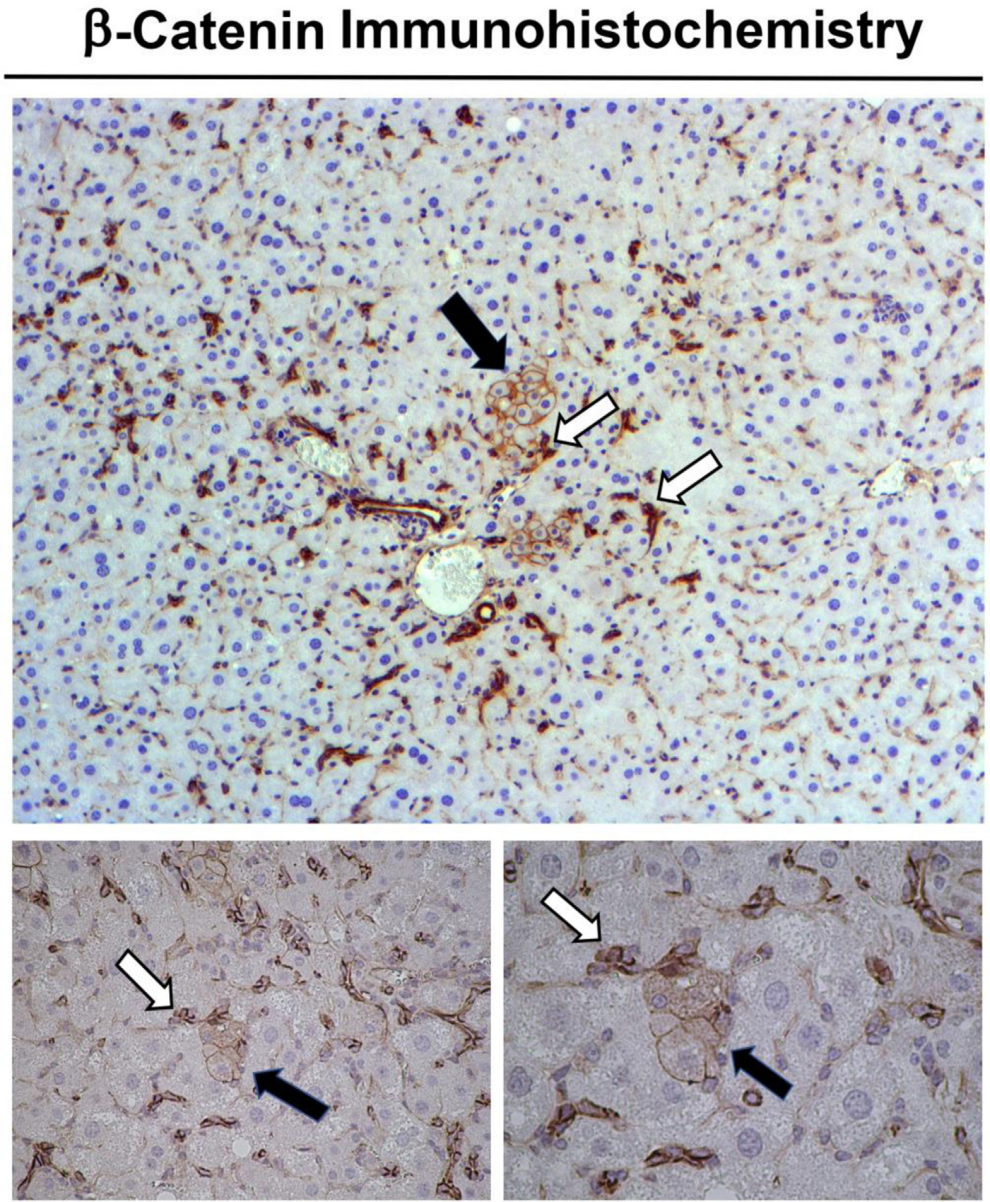
**Transdifferentiation of a subset of cholangiocytes within a ductular reaction into hepatocytes.** Immunohistochemistry for β-catenin shows appearance of β-catenin-positive small hepatocytes (*black arrows*) in close proximity to β-catenin-positive ductular reaction (*white arrows*) in the hepatocyte-specific β-catenin knockout mice after they were fed CDE diet for 2 weeks and switched to normal diet for 7 days for recovery. Original magnification x50 (*top*), x100 (*bottom left*), and x200 (*bottom right*).

A third way that ductular reaction may contribute to repair during chronic liver injury is the possibility that a subset of cholangiocytes within these structures could give rise to de novo hepatocytes. Hepatocytes, which are the major functional units of a liver, are often the main cell type afflicted by a multitude of insults. Normally, such injury results merely in death of some hepatocytes, allowing other hepatocytes to proliferate and replace the dying cells. However, if the injury is chronic and if hepatocyte proliferation is compromised because of senescence, epigenetic or signaling aberrations, or other mechanisms, hepatocyte markers begin to appear in a subset of cholangiocytes within a ductular reaction, demonstrating the likelihood of reprogramming and eventually transdifferentiation of such cholangiocytes into hepatocytes.^9^ This stochastic event has been shown to yield small hepatocytes that eventually proliferate, expand, and mature, to eventually contribute to hepatocyte repair as has been shown by careful fate-tracing studies.^9^, ^10^, ^11^ When hepatocyte-specific β-catenin knockout mice, which are incapable of normal hepatocyte proliferation, but with intact cholangiocyte β-catenin expression, have been administered CDE diet for 2 weeks followed by recovery on normal diet, a very small subset of β-catenin-positive cholangiocytes have been shown to give rise to β-catenin-positive small hepatocytes, which then proliferate and repopulate the liver (Figure 1).^10^^,^^12^ Such cell repopulation has been followed for several months after cessation of injury and has been found to be durable and nontransforming.^12^ Although heterogeneity in cholangiocytes is well documented, it is presently unclear whether only a small subset or all cholangiocytes possess comparable capability to transdifferentiate into hepatocytes, because both of these “hepithelial cells” are derived from a common progenitor during development.^13^, ^14^, ^15^ Because ductular reaction is often rampant in many clinical pathologies, which are primarily hepatocyte-directed, such as nonalcoholic steatohepatitis and alcoholic hepatitis, strategies to promote cholangiocyte-to-hepatocyte transdifferentiation through such mechanisms as epigenetic or signaling modulation may have notable clinical benefits.^13^^,^^16^

In conclusion, in a subset of liver pathologies, ductular reaction may have many beneficial effects, thus promoting the resilience and repair of this Promethean organ. Facilitating the process of ductular reaction-to-hepatocyte transdifferentiation through modulation of key signaling pathways may have notable therapeutic benefit in patients with hepatocyte insufficiency but presence of ample ductular reaction.
